# Die Aktivitäten des Resources Innovation Center im Spannungsfeld Brain Drain – Brain Circulation

**DOI:** 10.1007/s00501-021-01146-7

**Published:** 2021-09-16

**Authors:** Anna Meyer, Mariaelena Murphy

**Affiliations:** Resources Innovation Center Leoben, Montanuniversität Leoben, Leoben, Österreich

**Keywords:** Brain Drain, Brain Circulation, Osteuropa, Südosteuropa, Abwanderung, Migration, Konferenz, Rohstoffsektor, Brain drain, Brain circulation, East Europe, Southeast Europe, Migration, Conference, Raw material sector

## Abstract

Das Resources Innovation Center Leoben an der Montanuniversität Leoben veranstaltet seit 2014 eine Konferenzserie mit dem Titel ***E****ast and ****S****outh ****E****ast ****E****urope Dialogue Conferences. *Ende 2020 wurde die online Version der Konferenz genutzt um verschiedene Stakeholder des Rohstoffsektors, sowie auch Experten zum Thema Brain Drain zu Wort kommen zu lassen. Der Artikel umreißt das Spannungsfeld von Brain Drain und Brain Circulation und zeigt Möglichkeiten und zukünftige Schritte auf mit welchen man Kooperationen mit Ost- und Südosteuropa anstoßen und so zur Brain Circulation, dem multidirektionalen Fluss von Arbeitskräften und Wissen, beitragen kann.

## Abstract (en)

The Resources Innovation Center Leoben at the Montanuniversität Leoben has been organizing a conference series called *East and South East Europe Dialogue Conferences* since 2014. At the end of 2020, the online version of the conference was used to allow various stakeholders in the raw materials sector, as well as experts on the topic of brain drain, to have their say. The article outlines the tension between brain drain and brain circulation and shows possibilities and future steps to initiate cooperation with East and South East Europe and thus contribute to Brain Circulation, the multidirectional flow of manpower and knowledge.

## Die *East and South East Dialogue Conferences*

Das Resources Innovation Center (RIC) an der Montanuniversität Leoben ist seit Jahren erfolgreich darin, die Universität mit rohstoffrelevanten Akteur*innen und Institutionen zu verbinden. Dabei wurde seit 2014 und durch den 2015 erfolgten Gewinn der EIT RawMaterials Einreichung, die zum EIT RawMaterials mit MUL als Gründungsmitglied führte, ein Fokus auf den Südosteuroparaum gelegt. Seit dieser Zeit arbeitet das EIT RawMaterials Regional Center Leoben (EIT RC Leoben), als Teil des RIC, intensiv mit dieser Region. Dazu wurde eine Konferenzserie, die ***E****ast and ****S****outh ****E****ast ****E****urope Dialogue Conferences* (ESEE DC, [1]), ins Leben gerufen.

Die *ESEE Dialogue Conferences* zielen darauf ab, eine internationale Gemeinschaft im Rohstoffsektor zu fördern, indem sie relevante Stakeholder aus Industrie, Forschung und Bildung in Ost- und Südosteuropa zusammenbringen. Seit 2014 wurden in sieben Ländern zwölf Konferenzen zu verschiedensten Themen organisiert und dadurch viele Projekteinreichungen in Europäischen Förderprogrammen möglich gemacht.

Das Ergebnis der ESEE DC ist, dass die Montanuniversität Leoben (MUL) und das EIT RawMaterials in Kroatien, Slowenien, der Slowakei und Griechenland gut etabliert sind. Außerdem hat die MUL mit Serbien, Bosnien und Herzegowina, Montenegro, Nord-Mazedonien und Ungarn und Bulgarien im Rahmen des EIT RawMaterials zusammengearbeitet; Umstände, die einer hohen Anzahl von Projekten mit Partnern dieser Länder geführt haben. An den 52 genehmigten Projekten, die die MUL seit dem Beginn der Aktivitäten im EIT RawMaterials gewonnen hat, waren 32 Konsortien mit 19 verschiedenen Partnern aus der ESEE-Region beteiligt.

**Figure 1:**
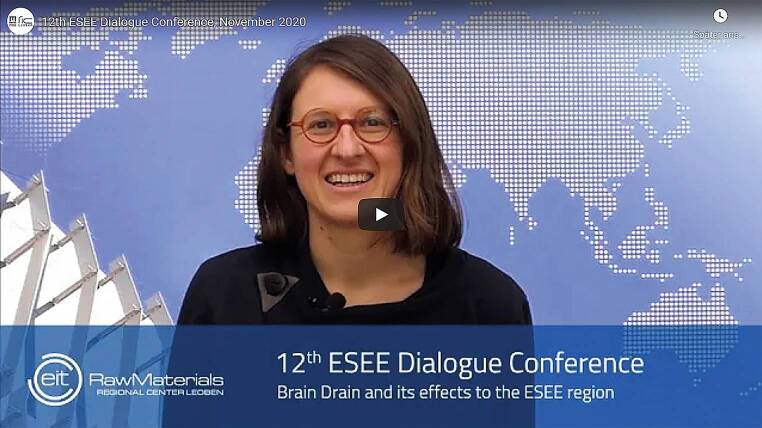

Bedingt durch die die COVID-19 Pandemie fand die *12. ESEE Dialoge Conference* im virtuellen Raum statt (Abb. 1). Diese neue Herausforderung brachte auch die Möglichkeit, das übliche lokale Set-Up, nämlich zwei ESEE-Länder pro Jahr mit einer tourenden ESEE-Dialogkonferenz zu besuchen, zu verlassen und die Teilnehmer*innen aus verschiedenen Ländern zu einem Thema zu verbinden. Ein Thema, das diesbezüglich hochspannend ist, ist die Abwanderung von hochgebildeten Arbeitskräften aus der Region. Daher wurde das Thema „Brain Drain und seine Auswirkungen auf die ESEE-Region“ als Thema der 12. ESEE DC gewählt.

Im Bericht des European Committee of Regions mit dem Titel *Addressing Brain Drain: The Local and Regional Dimension* wird Brain Drain wie folgt definiert: „[…] das Brain-Drain-Phänomen bezieht sich auf den dauerhaften Verlust von qualifizierten Arbeitskräften oder Studenten in einer Region. Die lokalen und regionalen Behörden in diesen Regionen müssen direkt mit den sozioökonomischen Auswirkungen fertig werden, die durch den erheblichen Verlust von Talenten verursacht werden“ [2]. Die Konferenz behandelte die Komplexität dieses Phänomens in mehreren Podiumsdialogen. Dabei wurde auf einen vielschichtigen Dialog, der Auswirkungen auf Bildung, Industrie und Politik und weitere Sektoren legt, geachtet. Mit dieser Konferenz eröffnete das RIC Leoben die Diskussion zu Brain Drain in der ESEE-Region sowohl im Rohstoffsektor (Tag 1) als auch in anderen Sektoren (Tag 2)^1^. Die Dialogkonferenzen sind dafür ein ideales Instrument, da sie sich als wertvoller erster Schritt erwiesen haben, um Diskussionen zu beginnen und neue Kooperationen zwischen Industrie, Wissenschaft und Forschung in Ost- und Südosteuropa zu schaffen. Mit dieser Konferenz hat das EIT RC Leoben seine Bemühungen auf die Diskussion der Auswirkungen auf den Brain Drain auf den Ebenen der Bildung, der Industrie und der Politik sowie auf unsere Mittel innerhalb des EIT und seiner KICs gelenkt. Im Wissen, dass Brain Drain weder sektor- noch länderspezifisch ist, hat das Zentrum die Konferenz für Teilnehmer aus allen Ländern und Sektoren geöffnet. Gerade beim Thema Brain Drain ist eine integrative und übergreifende Diskussion relevant und wichtig.

## Aspekte: Brain Drain – Brain Circulation

Anhand der Ergebnisse der Umfragen des Balkan Barometer [3], einer jährlichen Umfrage in sechs Ländern des Westbalkans (Albanien, Bosnien und Herzegowina, Kosovo, Montenegro, Republik Nord Mazedonien and Serbien) im Auftrag des Regional Cooperation Council, zeigen sich folgende relevante Daten:

Von den Befragten erwähnen 37 % ein Verlassen der Region (in Albanien sogar 46 % und in Montenegro 45 %), wobei 42 % es begrüßen, wenn Personen aus anderen Regionen in ihr Land kämen, um zu leben und zu arbeiten. Verbunden mit der Thematik des Brain Drain ist auch der in Aussicht gestellte Beitritt zu Europäischen Union dieser Länder. In den letzten fünf Jahren stieg die Einschätzung in der Region, dass der Beitritt zur EU eine ‚gute Sache‘ sei. Im Jahr 2021 sind 62 % der Befragten dieser Meinung. Hingegen ist immer noch unklar, wann dieser Betritt vorzogen sein wird da dies einstimmig von allen EU Mitgliedstatten beschlossen werden muss [4]. So hat zum Beispiel Nord Mazedonien den Beitrittskandidatenstatus seit 2005 [5]. Laut Balkanbarometer 2021 denken aber 31 % der Mazedonier*innen, dass es nie einen Beitritt geben wird. Generell glauben 40 % der Befragten der Westbalkanregion an einen Beitritt bis 2030, 24 % an einen Beitritt bis 2025 und 22 % glauben überhaupt nicht an einen Beitritt.

Vračić [6] argumentiert, dass eine Mitgliedschaft in der EU die Migration nicht ändert und nennt die Beispiele Bulgarien, Rumänien und Kroatien. Zusätzlich würden die Verzögerungen des EU-Beitritts die Frustration erhöhen und eine neue Welle der Migration befeuern. Daher argumentiert Vračić, dass ein Verständnis für die Implikationen der Emigration auf EU Seite aufgebaut werden muss. Was es braucht sei Dialog und die Bereitschaft Migrationsströme für beide Seiten zu nutzen, eine „circular migration“ [6].

Laut dem Balkanbarometer geben 63 % Prozent inadäquate Arbeitschancen als größtes Problem der jungen Menschen im Westbalkan an. Diese werden oft in anderen Ländern gesucht, und so kommt es zu hohen Abwanderungen von Fachkräften und gebildetem Personal. Dadurch ergeben sich eine Reihe von komplexen Problemen für die betroffenen Länder (hohe Investitionen in den Bildungssektor ohne Effekte, Verlust der Infrastruktur durch Arbeitskräftemangel, geringe Innovationskraft) Andererseits sind auch positive Effekte zu nennen wie Überweisungen aus der Diaspora [7].

Auch Vračić schärft den Blick auf das Phänomen des Brain Drain aus der Perspektive der Brain Circulation und hebt die positiven Aspekte der Diaspora in den Balkanländern hervor. Wichtig, um zu Lösungen und einem wirtschaftlichen wie sozialem Nutzen der Migrationsbewegungen zu kommen, sei ein Dialog innerhalb des Landes sowie auch ein Dialog mit den Zielländern und eine flächendeckende Erhebung von Migrationsbewegungen [6].

## Weiterführende Schritte

Durch die Konferenz wurden Wege aufgezeigt, wie sowohl durch Bildungsprojekte (OpESEE, [8] & LIMBRA, [9]) als auch durch Innovationsprojekte Mehrwert in der Süd- und Südosteuroparegion geschaffen werden kann. Wie eingangs erwähnt, ist dies auch ein erfolgreiches Betätigungsfeld des RIC an der MUL. Eine weitere Möglichkeit, um eine Zirkulation in beide Richtungen von Personen zu fördern, ist der Austausch von Studierenden. Ein solcher Austausch besteht zurzeit, soll nun aber intensiviert werden. Aktuell wird sondiert, wie sich die Montanuniversität im Spannungsfeld Brain Drain – Brain Circulation positionieren kann und aktiv ihre Beziehungen im Südosteuropäischen Raum nutzen kann um den pan-europäischen Gedanken weiter umzusetzen.

Der *Education and Training Monitor* 2019 [10] berichtet: „Lernmobilität ist mit zukünftiger Mobilität, höherem Verdienst und geringerer Arbeitslosigkeit verbunden. Sie korreliert auch mit verbessertem gegenseitigem Verständnis, Offenheit und staatsbürgerlichen Fähigkeiten^2^“ In diesem Sinne soll an der MUL ein Rahmenprogramm entstehen, das speziell den Austausch zwischen Studierenden aus Leoben und von Partneruniversitäten im Central European Exchange Program for University Studies (CEEPUS, [11]) Netzwerk fördert. Kern der Idee ist, dass neben den Lehrfächern Studierenden aus den ESEE Ländern ein Rahmenprogramm geboten werden soll, um ihre universitäre Ausbildung aus dem Heimatland zu komplettieren. Durch die Mobilität soll die Kooperation mit der Region vorangetrieben werden. Ziel ist es, langfristig die Beziehungen in diese Region aufrecht zu erhalten und zu verstärken.

Zurzeit wird ein Plan zur Umsetzung dieses Vorhabens erarbeitet und sondiert, mit welcher Universität ein Pilotprogramm aufgebaut werden soll. Der vorläufige Zeitplan sieht eine Einreichung im CEEPUS Netzwerk Ende 2021 vor.

Zusammenfassend ist das Thema Brain Drain ein brisantes und aktuelles für viele Wirtschaftssektoren in Süd- und Südost-Europa. Auch der Rohstoffsektor ist hier betroffen und Betriebe, Universitäten und einzelne Akteure*innen haben dies erkannt und arbeiten an verschiedenen Projekten, die die Region stärken, in Projekte einbinden und Perspektiven schaffen.

Nicht alles kann von der Seite dieser Akteure *innen geleistet werden, und so muss auch die Politik die geplanten Erweiterungen durchführen. Durch die 12. ESEE Dialogue Conference wurde jedoch klar, dass sich die Akteure*innen des Rohstoffsektors diesem Thema angenommen haben und es auf Initiative des RIC Leoben zu weiterführenden Aktivitäten kommen wird.

## Footnotes

1 Details und Konferenzmitschnitte unter https://esee-dc.eu/past-events/.

2 Originalzitat auf Englisch.
